# Evaluation of cartilaginous endplate degeneration with histogram features of multiple parameters in UTE MRI

**DOI:** 10.1186/s12880-026-02413-0

**Published:** 2026-05-15

**Authors:** Jingwei Miao, Chuanyan Li, Yu-Xuan Ren, Bingcang Huang

**Affiliations:** 1 Public College of Medical Technology, University of Shanghai for Science and Technology, Shanghai, 200093 China; 2 Department of Radiology, Gongli Hospital, Pudong, Shanghai, 200135 China; 3 Jinshan Hospital, Institute for Translational Brain Research, Fudan University, Shanghai, 200032 China

**Keywords:** Ultrashort echo time, Cartilage endplate, Bi-exponential $T_2^*$ model, Degeneration grading

## Abstract

**Objective:**

The cartilaginous endplate (CEP) exhibits an intrinsically short $T_2^*$ relaxation time, making it difficult to visualize using conventional MRI sequences. This limitation hampers early detection and quantitative evaluation of lumbar spine degeneration. This study aimed to perceive microstructural alterations of CEP at early stages of degeneration using a bi-exponential $T_2^*$ model based on ultrashort echo time (UTE) sequence, and to explore the diagnostic utility of bi-exponential $T_2^*$–derived parameters and monoexponential UTE-$T_{2}$ mapping values in grading CEP damage.

**Methods:**

This study retrospectively collected 46 patients who underwent lumbar UTE MRI, of whom 43 met the inclusion criteria. CEP was graded into three groups according to morphological features on multiple MRI sequences: healthy (structurally intact), mild damage (localized thinning or concavity with preserved continuity), and moderately damage (defects < 50% with disrupted continuity). Multiple quantities were evaluated using the UTE sequence on the CEP manually drawn by an experienced radiologist. One-way Kruskal-Wallis test was used to inspect the distribution differences among groups. Logistic regression and support vector machine models were applied to predict the level of degeneration with a considerably good precision, and receiver operating characteristic curves suggests a distinguishable performance among those models.

**Results:**

$T_{2S}$ values showed significant differences among the groups (*p* < 0.05), with Tukey’s test indicating the most significant difference between the moderately damaged group and the healthy controls. $\:{T}_{2L}$ values were non-normally distributed but statistically different between the moderate and mild damage groups (*p* < 0.05), a trend also observed in monoexponential UTE-$T_{2}$ mapping values (*p* < 0.05). The logistic regression and SVM models performed well in identifying moderate damage (AUC of 0.878 and 0.858, respectively), but had limited ability to detect mild damage (AUC of 0.718 and 0.729).

**Conclusion:**

The UTE bi-exponential $T_2^*$ model enable effective separation and quantification of distinct water components within the CEP. Both $\:{T}_{2L}$ and UTE-$T_{2}$ mapping show promise as imaging biomarkers for grading CEP degeneration even in the early stage of CEP degeneration.

## Introduction

The human vertebrate mainly comprises vertebrate body and the intervertebral disc (IVD). There exists a thin layer of hyaline cartilage (~ 1 mm thick) between the vertebrate body and the IVD [1]. Coined as cartilaginous endplate (CEP), this thin layer of tissue is crucial to maintain the intervertebral height and transmit the mechanical load among vertebrates [2]. The CEP also plays a pivotal role in sustaining disc homeostasis, since the vascular-free IVD relies on the CEP to transport nutrients and eliminate the metabolic wastes [3]. When the CEP is thinned or disrupted, nutrient transport to the IVD may be impaired, ultimately leading to disc degeneration [4]. The magnetic resonance imaging (MRI) relies on the water diffusion to map tissue structure. The cartilaginous endplate is characterized by a relatively short transverse relaxation time, which was reported to be in a broad range from approximately 9.9 to 21.8 ms [5], depending on tissue composition, hydration status, and the degree of degeneration [6]. However, the bound water contribution change in CEP has not been well understood in the evolution of IVD degeneration, since the bound water contribution of CEP exhibits considerably low signal on conventional $\:{T}_{2}$-weighted images even if it is fractured or defective.

The ultrashort echo time (UTE) sequence collects magnetic resonance signal with an ultrafast echo time on the order of microsecond before the rapid decay of short $\:{T}_{2}$ tissues. Thus, it enables effective imaging of short $\:{T}_{2}$ tissues that are difficult to detect with conventional MRI, such as bone, tendons, cartilage, and ligaments [7, 8]. High signal intensity near the CEP was found on 2D-UTE subtraction image morphology to distinguish the calcified and uncalcified CEP [9]. UTE image has identified the correlation between CEP lesions and IVD degeneration in patients with chronic low back pain [10]. While current research focuses on evaluating morphological and pathological changes of lumbar CEP, it is still challenging to quantitatively assess the minute change in the early stage of CEP degeneration. In particular, precise analysis of changes in bound and free water components following early CEP damage is lacking, which severely hampers early diagnosis of lumbar degenerative diseases [6].

Bi-component $\:{T}_{2}^{*}$ model has been widely applied to separate bound and free water components. The $\:{T}_{2}^{*}$ of bound water is approximately ten times shorter than that of free water, and they can be separated through UTE sequence combined with bi-exponential analysis [11, 12]. Therefore, we quantitatively analyze lumbar CEP using a bi-exponential $\:{T}_{2}^{*}$ model by extracting the decay curves of short and long $\:{T}_{2}^{*}$ components to accurately identify the CEP degeneration grade. This provides reliable evidence for early identification and quantitative evaluation of CEP damage, and thus offers theoretical support for early diagnosis and intervention strategies for chronic low back pain and progressive disc diseases.    

## Methods

This study was approved by the institutional ethics committee (Approval No. GLYY1s2025-091), and all procedures were conducted in accordance with the principles of the Declaration of Helsinki. Given the retrospective design and the de-identified nature of the data, the requirement for informed consent was waived by the ethics committee.

### Study population

**Fig. 1:**
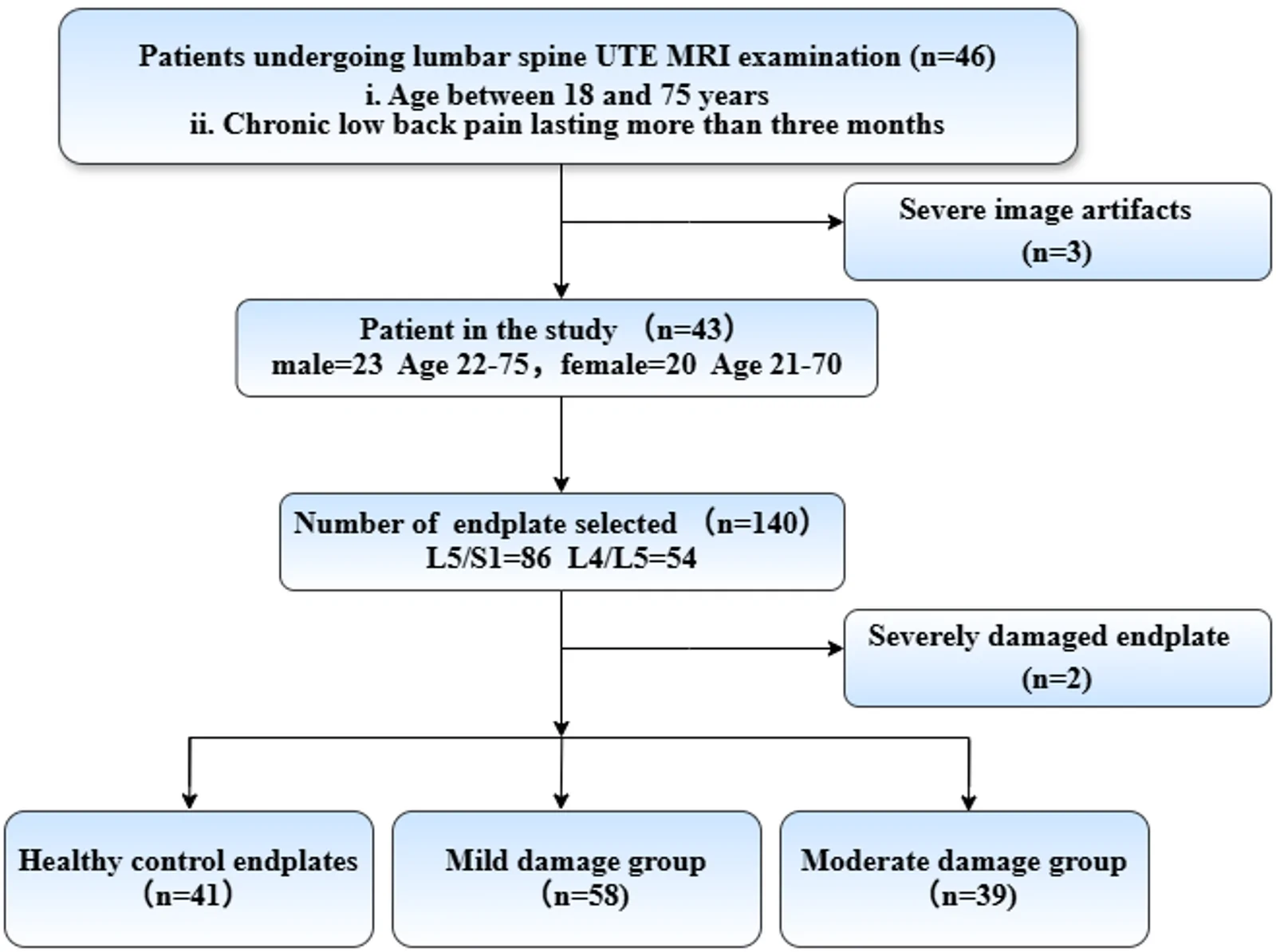
Flow chart of patient screening and endplate grouping. Forty-six patients who underwent UTE MRI of the lumbar spine were initially enrolled, and after excluding three cases due to severe image artifacts, 43 patients (23 males, 20 females; age range 21–75 years) were finally included. A total of 140 lumbar endplates (86 in the L5/S1 segment and 54 in the L4/L5 segment) were selected for evaluation. Damaged endplates were categorized into three groups based on morphological characteristics: a healthy endplate group (*n* = 41), a mildly damaged group (*n* = 58), and a moderately damaged group (*n* = 39). Two severely damaged endplates were excluded from the subgroup analysis

In this study, 46 patients who underwent lumbar UTE MRI at Gongli Hospital, Pudong New District, Shanghai, China, from July 2022 to November 2023 were retrospectively collected. Patient inclusion and exclusion criteria were shown in Fig. 1. Briefly, the inclusion criteria were (1) age 18–75 years old, (2) lower back pain symptoms lasting more than three months, and (3) complete MRI imaging data. The exclusion criteria included (1) history of previous acute lumbar trauma or surgery; (2) vertebral fracture or compression lesion; (3) suffering from specific diseases such as spinal deformity or ankylosing spondylitis; (4) poor image quality, which affects the endplate identification and outlining; and (5) severe CEP damage. After screening, 43 patients were finally enrolled, including 23 males and 20 females.

### Image processing and VOI segmentation

All MR scans were done on a 3.0 T MR scanner (Vantage Titan, Canon Medical System, Japan) using a 16-channel dedicated spine coil. In order to accurately assess the signal characteristics of the different $\:{T}_{2}^{*}$ components in the CEP, images of the endplate region were acquired using a three-dimensional four-echo UTE sequence, with the following scanning parameters: the repetition time (TR) was 14.3 ms, the four echo times were $\:T{E}_{1}$=0.096 ms, $\:T{E}_{2}$=2.7 ms, $\:T{E}_{3}$=5.3 ms and $\:T{E}_{4}$=7.9 ms, the flip angle was $\:{10}^{\circ\:}$, the receiver bandwidth was 488 Hz/pixel, the field of view (FOV) was 380 × 380 mm, the matrix was 512$\:\times\:$512, and the layer thickness was 2 mm.

The fifth lumbar vertebrae and first sacral vertebrae are the lowermost part of the human spine, which bear the main load from the upper body and are most prone to degeneration and damage. Therefore, the L5 lower CEP and the S1 upper CEP of all patients were preferred as the initial analysis area, and the complete CEP was labeled as the volume of interest (VOI). A total of 86 VOIs were obtained. Because most of the CEP degeneration in the L5-S1 segment was mild to moderate, in order to expand the distribution of degeneration levels and improve the statistical efficacy, 27 VOIs from the 43 subjects were selected from the Lumbar 4 sublaminar CEP and Lumbar 5 supraspinal CEP, and 54 additional endplate VOIs were included. In total, 138 endplate VOIs were enrolled for imaging analysis and quantitative evaluation.

### Grading and grouping of cartilage endplates

In clinical practice, Modic classification is primarily used to evaluate bone marrow signal changes beneath the cartilage endplate, reflecting bone marrow reactivity secondary to endplate pathology, rather than the structural integrity of the cartilage endplate itself [13]. To specifically assess cartilage endplate structural damage, we adopted the optimized CEP classification system proposed by Rajasekaran et al., which is based on UTE-MRI imaging features and focuses on the continuity, integrity, and defect patterns of the CEP [10]. This typing method was used to categorize CEP into three groups in this study:

1. Patients with structurally intact CEP and good continuity were defined as healthy controls.
2. Patients with partial or total thinning or depression of the CEP, but with good continuity, were defined as the mildly degenerated group.
3. Patients with CEP defects covering less than $\:50\%$ of the surface area, as well as impaired structural continuity, were defined as the moderately damaged group.

Patients with severe damage to the CEP exceeding $\:50\%$ of the area were excluded because this study aimed to identify imaging features of early CEP degeneration. All CEP grading were performed independently by two radiologists with more than five years of experience in diagnostic spine imaging. The radiologists were unaware of any clinical information. In case of disagreement, the final grade was adjudicated by a third expert with over 20 years of experience. To further ensure the robustness of CEP grading, inter- and intra-observer agreement analyses were performed. A subset of cases was independently graded by both radiologists for inter-observer assessment, and repeated grading was conducted by the primary reader after a washout period for intra-observer evaluation. Inter- and intra-observer agreement were quantified using Cohen’s kappa statistics. All grading operations were performed on the original UTE images with $\:{T}_{1}$- and $\:{T}_{2}$- weighted images are as reference.

### Data process

All images were exported from the Picture Archiving and Communication System (PACS) in Digital Imaging and Communications in Medicine (DICOM) format. The DICOM images are further converted into the Neuroimaging Informatics Technology Initiative (NifTi) format for easy handling and processing. The CEP was identified based on the original UTE images and subtraction images. The VOI was manually drawn with the ITK-SNAP software (Version 4.2.2, www.itksnap.org) on the first UTE image and further duplicated on subsequent images. All VOIs were manually delineated on UTE images by a radiologist with more than five years of experience in spine imaging, following a standardized segmentation protocol. Given the thin and irregular morphology of the CEP, manual delineation was chosen to ensure accurate anatomical localization, and all VOIs were subsequently reviewed and confirmed by a second radiologist to minimize potential observer-related bias. Quantitative parameter extraction and subsequent statistical and machine learning analyses were performed in a fully objective manner. Biexponential fitting was performed using MATLAB (Matlab 2025a, The MathWorks Inc., Natick, MA, USA) across all the pixels within the VOI including intact and injured sites, and the bi-component model reads [14],

1 $$\:S\left(t\right)/{S}_{0}=f\times\:{exp}\left(-\frac{t}{{T}_{2S}^{*}}\right)+(1-f)\times\:{exp}\left(-\frac{t}{{T}_{2L}^{*}}\right)+noise$$

where the fitted constant noise term accounts for background noise, * f* represents the ratio of the bound water component, and $\:{T}_{2S}^{*}$ and $\:{T}_{2L}^{*}$ are respectively the short and long $\:{T}_{2}^{*}$ relaxation times. In addition to bi-exponential $\:{\mathrm{T}}_{\mathrm{2}}^{\mathrm{*}}$ analysis, monoexponential fitting of the UTE signal decay was performed to derive an apparent transverse relaxation time, referred to as UTE-$\:{\mathrm{T}}_{\mathrm{2}}^{\mathrm{*}}$. This parameter reflects the effective $\:{\mathrm{T}}_{\mathrm{2}}^{\mathrm{*}}$ behavior of the cartilage endplate without explicit separation of bound and free water components and was included for comparison with bi-exponential parameters.

### Statistical analysis

All statistical analyses were done using MATLAB and the data assessed for normality by the Shapiro-Wilk and expressed as mean $\:\pm\:$ standard deviation (SD) or median and interquartile range (IQR) as appropriate. Differences were assessed using the Wilcoxon, one-way ANOVA, and Tukey’s post-hoc tests. The Kruskal-Wallis test was used for non-Gaussian data. For predictive modeling, logistic regression (LR) and support vector machine (SVM) classifiers were constructed using quantitative UTE-derived parameters. Given the limited sample size, model performance was evaluated using stratified k-fold cross-validation to ensure robust estimation while preserving class balance across folds. All quantitative parameters were standardized before model training. To reduce the risk of overfitting, model complexity was constrained and no outcome-driven feature selection was performed outside the cross-validation framework. Model performance was assessed using receiver operating characteristic (ROC) analysis, with the area under the curve (AUC), sensitivity, and specificity calculated for each model. 95% confidence intervals (95% CIs) for AUC values were estimated using a non-parametric bootstrap resampling method. A two-sided p value < 0.05 was considered statistically significant.

## Results

### Clinical information

This study included a total of 43 patients, with a mean age of $\:46.8\pm\:15.0$ years and an interquartile range of 35.3 to 58.0 years. The Shapiro-Wilk test revealed that the age distribution did not significantly deviate from normal (h = 0, *p* = 0.16). The mean age was $\:46.2\pm\:15.9$ years for males (* N=23*, range: 22.0–75.0 years, IQR: 34.0–57.8) and $\:47.5\pm\:14.2$ years for females (* N=20*, range: 21.0–70.0 years, IQR: 39.0–58.0). The rank sum test revealed no significant difference in age between males and females (*p* = 0.62) or between males and the entire group, as well as between females and the entire group (*p* = 0.78; *p* = 0.76).

Inter-observer agreement for CEP grading demonstrated good consistency, with a weighted Cohen’s kappa of 0.829 (95% CI: 0.762–0.897). Inter-observer agreement was higher, indicating good reproducibility of the grading procedure. Figure 1 illustrates the entire screening and grading criteria, comprising 41 CEPs in the healthy control group, 58 CEPs in the mildly damaged group, and 39 CEPs in the moderately damaged group.

**Fig. 2:**
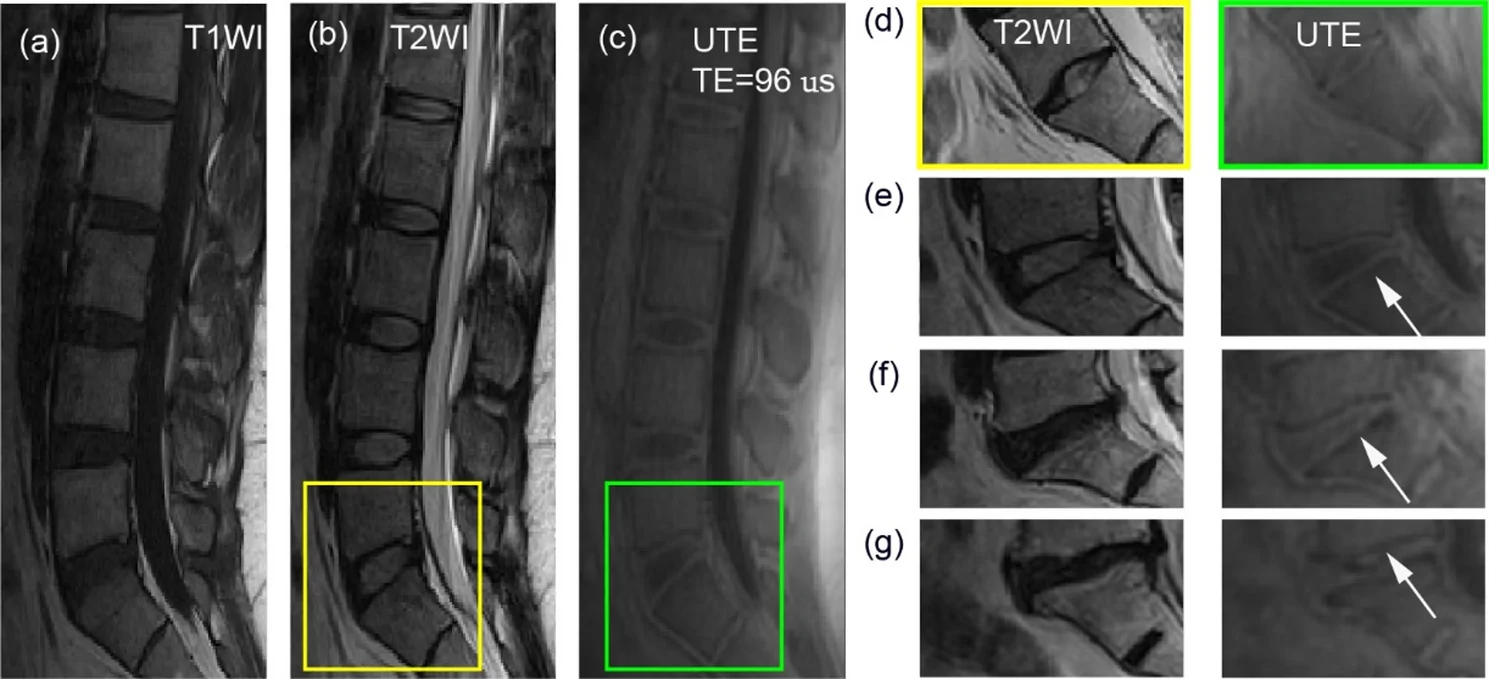
Comparison of Conventional versus Ultrashort Echo Time (UTE) Sequences in Lumbar Sagittal Magnetic Resonance Imaging of the Cartilage Endplate. The cartilage endplate signal is weak and difficult to recognize in (**a**) the T1-weighted image (T1WI) and (**b**) the $\:{\mathrm{T}}_{2}$-weighted image (T2WI). (**c**) Images obtained using UTE (TE = 96 ms) show the cartilaginous endplates of the lower lumbar segments with markedly high signals. Images (**d–g**) show the cartilaginous endplates in the L5–S1 region. The white arrowheads indicate areas of the endplates that are not visible in conventional sequences but show a high signal in UTE images. The left column is the T2WI image, and the right column is the corresponding UTE image

The CEP region was analyzed using a biexponential fitting on three groups of UTE images (Fig. 2). Figure 2a-c shows representative sagittal MR images of the lumbar spine from a 44-year-old male patient with chronic low back pain, clinically diagnosed with lumbar disc degeneration. Conventional sequences included (a) $\:{\mathrm{T}}_{\mathrm{1}}$-weighted imaging ($\:{\mathrm{T}}_{\mathrm{1}}$WI) and (b) $\:{\mathrm{T}}_{\mathrm{2}}$-weighted imaging ($\:{\mathrm{T}}_{\mathrm{2}}$WI), which clearly depicted the vertebral bodies, intervertebral discs, and endplate morphology. The yellow box in (b) marks the L5–S1 region of interest. (c) A UTE image (TE = 96µs) was acquired to capture signal from short-$\:{\mathrm{T}}_{\mathrm{2}}$ tissues such as the cartilage endplate, with the green box indicating the same anatomical region as in (b). Figure 2(d–e) shows the L5–S1 CEP in a healthy control subject. The left column displays $\:{\mathrm{T}}_{\mathrm{2}}$WI images, while the right column shows UTE images, in which the cartilage endplate structure is depicted more clearly than in conventional $\:{\mathrm{T}}_{\mathrm{2}}$WI. White arrows indicate the location of the cartilage endplate, where the short-$\:{\mathrm{T}}_{\mathrm{2}}$ signal is more prominent in the UTE images.

### Intergroup comparison of bi-exponential $\:{\mathrm{T}}_{\mathrm{2}}^{\mathrm{*}}$ parameters

**Fig. 3:**
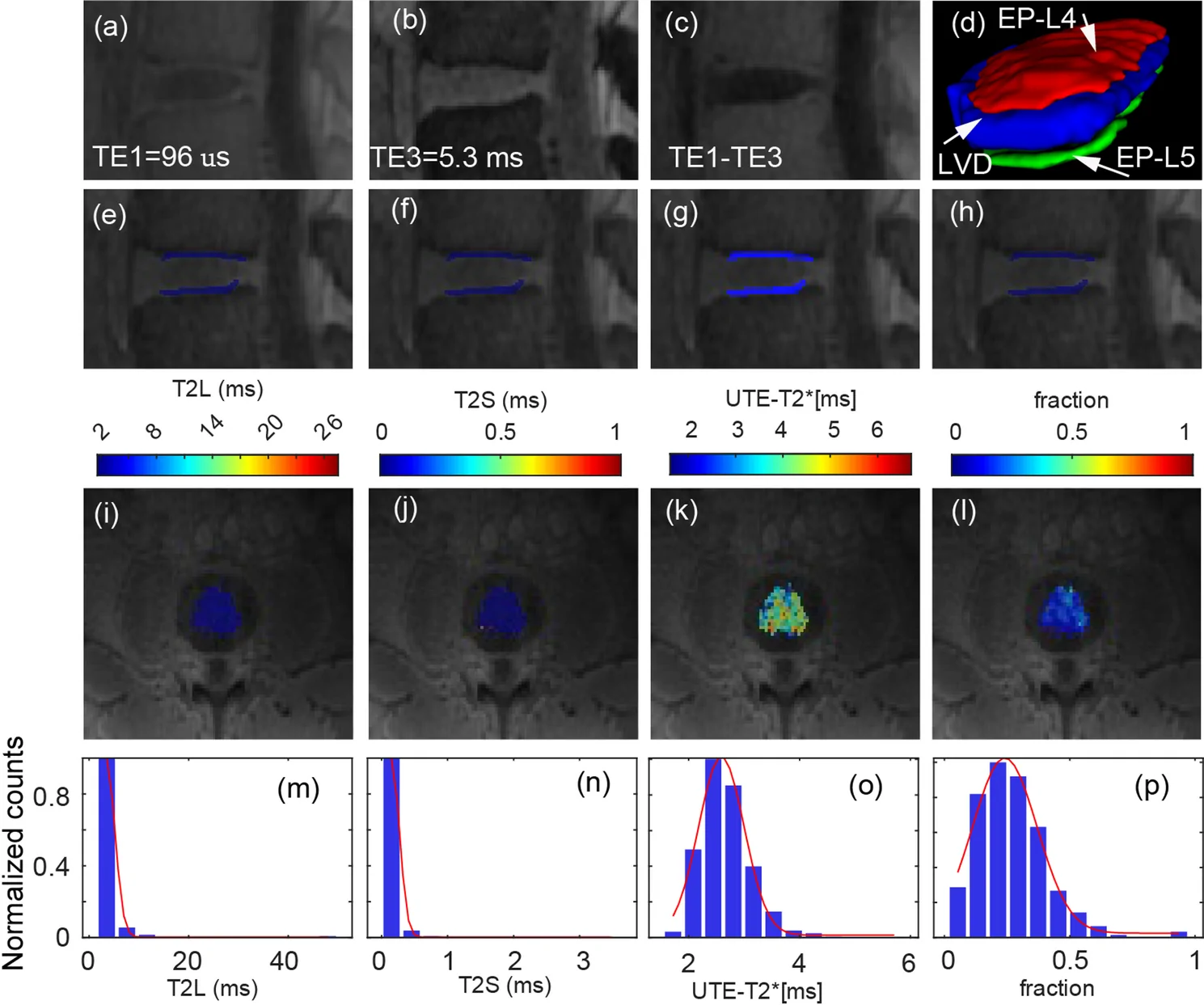
Multi-parameter visualization analysis for quantitative assessment of cartilage endplates by ultrashort echo time (UTE) bi-exponential $\:{\mathrm{T}}_{2}^{\mathrm{*}}$ imaging. (**a-c**) Sagittal UTE images and subtracted images at different echo times, showing the morphology of the cartilage endplates. (**d**) Schematic of 3D reconstruction, including the cartilage endplates of L4 and L5 segments (EP-L4, EP-L5) and the intervertebral disc space (LVD). (**e-h**) Sagittal ROI outlining schematic of UTE images and subtracted images at different echo times. (**i-l**) The corresponding post-fitting cross-sectional parameter maps show: (**i**) long $\:{\mathrm{T}}_{2}$ component ($\:{\mathrm{T}}_{2\mathrm{L}}$), (**j**) short $\:{\mathrm{T}}_{2}$ component ($\:{\mathrm{T}}_{2\mathrm{S}}$), (**k**) bi-exponential UTE-$\:{\mathrm{T}}_{2}$ value, and (**l**) short $\:{\mathrm{T}}_{2}$ component percentage (fraction). (**m-p**) Histograms of $\:{\mathrm{T}}_{2\mathrm{L}}$,$\:{\mathrm{T}}_{2\mathrm{S}}$,UTE-$\:{\mathrm{T}}_{2}^{\mathrm{*}}$,and fraction distributions, with red lines representing the fitted model

The analysis extracted the short $\:{\mathrm{T}}_{\mathrm{2}}^{\mathrm{*}}$ ($\:{\mathrm{T}}_{\mathrm{2}\mathrm{S}}$), long $\:{\mathrm{T}}_{\mathrm{2}}^{\mathrm{*}}$ ($\:{\mathrm{T}}_{\mathrm{2}\mathrm{L}}$), UTE-$\:{\mathrm{T}}_{\mathrm{2}}^{\mathrm{*}}$ mapping, and $\:{\mathrm{T}}_{\mathrm{2}\mathrm{S}}$ fraction parameters for all voxels in the VOI. These parameters reflected the tissue properties of the CEP under different injury levels (Fig. 3). The short-TE UTE image (Fig. 3a, TE1 = 96 µs) provided high signal from short-T2 tissues, while the long-TE image (Fig. 3b, TE3 = 5.3 ms) showed attenuated signal in these tissues. The subtraction image (Fig. 3c, TE1 − TE3) highlighted the short-$\:{\mathrm{T}}_{\mathrm{2}}$ components. The 3D VOI (Fig. 3d) was delineated to include the CEP at the EP-L4 and EP-L5 levels and the adjacent lumbar vertebral disc (LVD). Parametric maps derived from bi-component analysis included the long-$\:{\mathrm{T}}_{\mathrm{2}}$ ($\:{\mathrm{T}}_{\mathrm{2}\mathrm{L}}$) map (Fig. 3e), short-$\:{\mathrm{T}}_{\mathrm{2}}$ ($\:{\mathrm{T}}_{\mathrm{2}\mathrm{S}}$) map (Fig. 3f), UTE-$\:{\mathrm{T}}_{\mathrm{2}}^{\mathrm{*}}$ mapping (Fig. 3g), and the short-$\:{\mathrm{T}}_{\mathrm{2}}$ fraction map (Fig. 3h). Overlays of these maps on the anatomical images (Fig. 3i–l) facilitated visualization of the spatial distribution of each parameter within the CEP. The histograms of each quantitative parameter (Fig. 3m-p) further reveal the tissue characteristics of different regions of the cartilage endplate. The distributions of $\:{\mathrm{T}}_{\mathrm{2}\mathrm{L}}$, $\:{\mathrm{T}}_{\mathrm{2}\mathrm{S}}$, and UTE-$\:{\mathrm{T}}_{\mathrm{2}}^{\mathrm{*}}$ mapping all exhibit right-skewed distributions, indicating that most regions have lower $\:{\mathrm{T}}_{\mathrm{2}}$ values concentrated in the shorter echo time range.

First, normality was tested using the Shapiro-Wilk test to assess the distribution characteristics of each parameter. The results showed that the $\:{T}_{2S}$ and $\:{T}_{2S}$ fraction parameters were normally distributed. The $\:{T}_{2L}$ and UTE-$\:{\mathrm{T}}_{\mathrm{2}}^{\mathrm{*}}$ mapping parameters did not conform to a normal distribution. 

The results showed that the mean $\:{T}_{2S}$ values for the healthy control, mild damage, and moderate damage groups were $\:0.20\pm\:0.06$ ms, $\:0.22\pm\:0.08$ ms, and$\:\:0.25\pm\:0.11\:$ms, respectively. One-way analysis of variance revealed statistically significant differences among the three groups (*p* < 0.05), and the Tukey post hoc test showed significant differences between the healthy control group and the other two groups. The $\:{T}_{2L}$ values were non-normally distributed, with median $\:{T}_{2L}$ values of 7.4 ms (IQR: 4.6–11.9 ms), 8.7 ms (IQR: 5.7–11.1 ms), and 5.3 ms (IQR: 4.3–8.0 ms) in the healthy control, mildly impaired, and moderately impaired groups, respectively. The inter-group difference analysis revealed a statistically significant difference only between the moderate and mild damage groups (*p* < 0.05). This suggests that $\:{T}_{2L}$ values can differentiate between groups when assessing the severity of endplate damage.

**Fig. 4:**
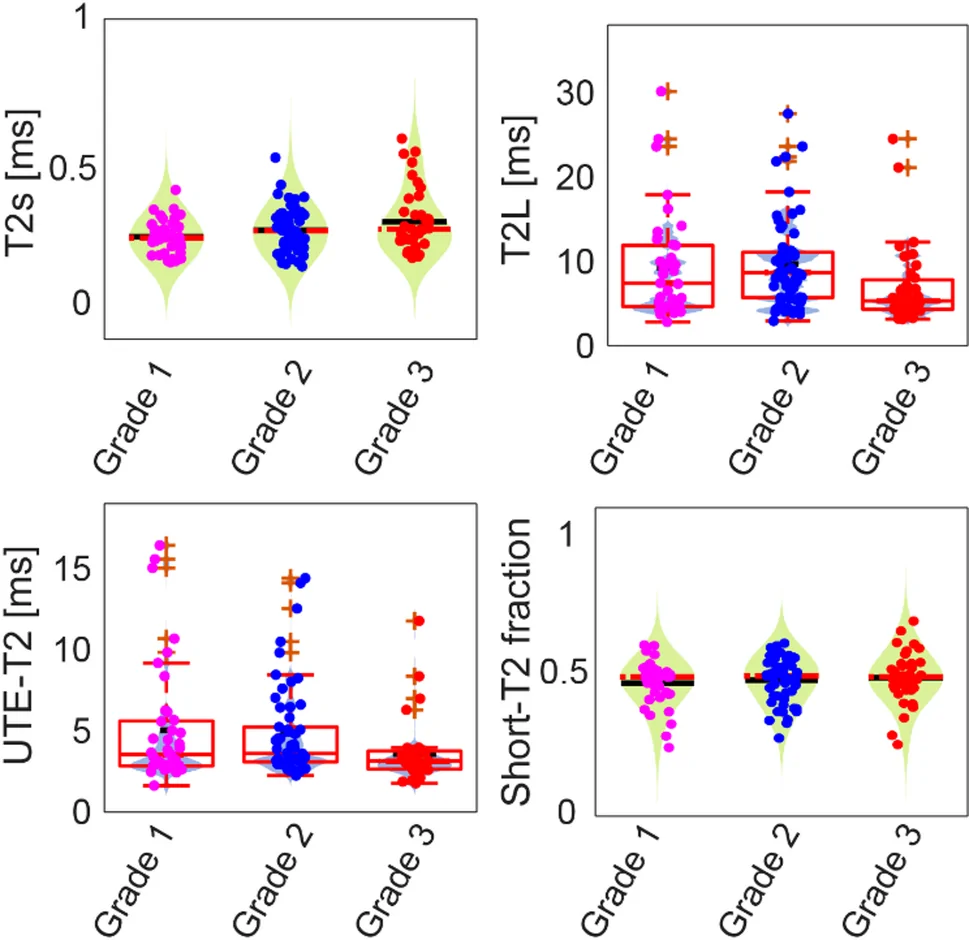
Comparison of $\:{\mathrm{T}}_{2}$ Component Parameters of Cartilage Endplates Between Different Groups (Top left) Short $\:{\mathrm{T}}_{2}$ fraction ($\:{\mathrm{T}}_{2\mathrm{S}}$, ms), obtained by UTE bi-exponential model fitting. (Top right) Long $\:{\mathrm{T}}_{2}$ fraction ($\:{\mathrm{T}}_{2\mathrm{L}}$, ms), shown as box plots and scatter plots. (Bottom left) $\:{\mathrm{T}}_{2}$ values (ms), obtained by conventional UTE-$\:{\mathrm{T}}_{2}$ mapping. (Bottom right) Proportion of the T2S fraction in the total signal ($\:{\mathrm{T}}_{2\mathrm{S}}$ fraction). Violin plots show the density of the data distribution, with the black horizontal line indicating the median. The pink, blue, and red colors represent the healthy control, mild damage, and moderate damage groups, respectively

UTE-$\:{\mathrm{T}}_{\mathrm{2}}^{\mathrm{*}}$ mapping values also showed a decreasing trend. The median values for the three groups were 3.5 ms (IQR: 2.8–5.6 ms), 3.6 ms (IQR: 3.1–5.4 ms), and 3.2 ms (IQR: 2.6–3.8 ms). The Kruskal–Wallis test showed a significant difference between the moderate and mild injury groups. The $\:{T}_{2S}$ fraction reflects the proportion of $\:{T}_{2S}$ components in the total signal. The mean $\:{T}_{2S}$ fraction values in the three groups were $\:0.32\pm\:0.09$, $\:0.33\pm\:0.08$, and $\:0.34\pm\:0.10$, respectively. One-way ANOVA showed that the difference between groups was not significant (*p* = 0.58), suggesting that this index weakly differentiates the degree of endplate damage (Fig. 4).

### Classification of CEP degeneration level with quantitative bi-component UTE features

**Fig. 5:**
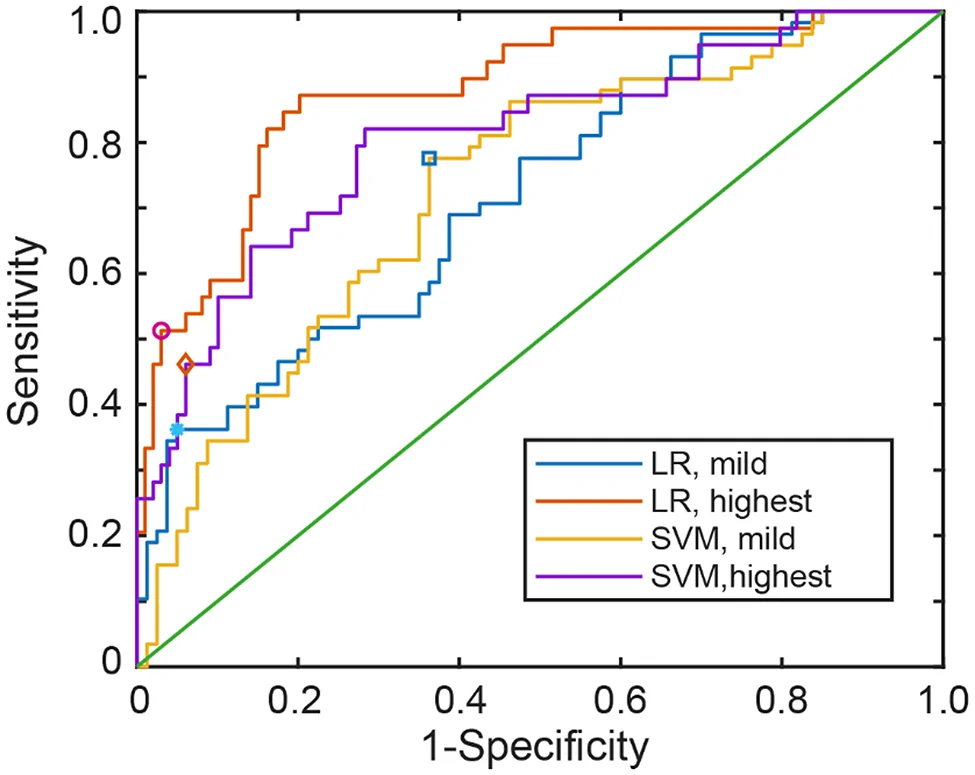
Receiver operating characteristic (ROC) curves of logistic regression versus support vector machine (SVM) models for classifying intervertebral endplates with different levels of degeneration. It shows the performance of the logistic regression model (blue and orange) versus the SVM model (yellow and purple) in classifying mild versus moderate endplate damage. The horizontal axis is the false positive rate, and the vertical axis is the true positive rate. The diagonal line indicates random classification performance

Further ROC curve corroborates the diagnostic efficacy of the above MRI parameters in different endplate damage levels. Figure 5 shows the ROC curves of the Logistic Regression (LR) and Support Vector Machine (SVM) models, which were constructed based on UTE bi-exponential parameters, for predicting different endplate damage grades. The study conducted ROC curve analysis using two models (LR and SVM) with 16 quantitative parameters. These parameters included: $\:{T}_{2S}$ mean, $\:{T}_{2S}$ standard deviation, $\:{T}_{2S}$ 25th percentile, $\:{T}_{2S}$ 75th percentile, $\:{T}_{2L}$ mean, $\:{T}_{2L}$ standard deviation, $\:{T}_{2L}$ 25th percentile, $\:{T}_{2L}$ 75th percentile, $\:{\mathrm{T}}_{\mathrm{2}}^{\mathrm{*}}$ mapping, $\:{\mathrm{T}}_{\mathrm{2}}^{\mathrm{*}}$ mapping standard deviation, $\:{\mathrm{T}}_{\mathrm{2}}^{\mathrm{*}}$ mapping 25th percentile, $\:{\mathrm{T}}_{\mathrm{2}}^{\mathrm{*}}$ mapping 75th percentile, $\:{T}_{2S}$ fraction, $\:{T}_{2S}$ fraction standard deviation, $\:{T}_{2S}$ fraction 25th percentile, and $\:{T}_{2S}$ fraction 75th percentile. The results showed that both models performed better in predicting moderate injury than mild damage.

For predicting moderate endplate injuries, the LR model achieved the best results, with an AUC value of 0.878 (95% CI: 0.794–0.931). The SVM model also performed similarly in this group, with an AUC of 0.806 (95% CI: 0.713–0.879). The performance of both models decreased in the prediction of mild damage, with an AUC of 0.718 (95% CI: 0.624–0.976) for LR and 0.729 (95% CI: 0.643–0.802) for SVM.

## Discussion

We systematically evaluated tissue change of CEP under different injury grades using a bi-exponential $\:{T}_{2}^{*}$ model. We verified the diagnostic efficacy of $\:{T}_{2S}$, $\:{T}_{2L}$, UTE-$\:{T}_{2}^{*}$ mapping, and $\:{T}_{2S}$ fraction parameters in grading CEP damage by constructing two prediction models: a LR model and a SVM model. The results showed that $\:{T}_{2S}$ and $\:{T}_{2L}$, which reflect the content of the water component of cartilage tissue and the structure of the collagen matrix, were highly sensitive to moderate CEP damage.

The $\:{T}_{2S}$ value reflects tthe relaxation behavior of water molecules tightly associated with the collagen network and is sensitive to collagen density, alignment, and macromolecular packing [15]. One-way ANOVA revealed statistically significant differences in $\:{T}_{2S}$ values among groups (*p* < 0.05). The Tukey post hoc test showed that the $\:{T}_{2S}$ value in the moderate CEP damage group was significantly higher than in the healthy group (*p* < 0.05). The significant increase in $\:{T}_{2S}$ observed in the moderately damaged group suggests microstructural disorganization of the collagen matrix, leading to altered bound water relaxation. Previous studies have reported that the $\:{T}_{2S}$ component increases significantly in degenerative cartilage without significant changes in $\:{T}_{2L}$ [16]. Degenerative tendons also lead to increased $\:{T}_{2S}$ fractions [17]. All of these studies demonstrated the high sensitivity of $\:{T}_{2S}$ to early microstructural changes.

The Kruskal-Wallis test was used to compare the difference in $\:{T}_{2L}$ values between groups. The results showed that the $\:{T}_{2L}$ values of the moderately impaired group were significantly lower than those of the mildly impaired group (*p* < 0.05). This suggests that free water content decreases with an increase in CEP damage, which may be a result of decreased tissue permeability [18]. Higher degrees of disc degeneration results in lower $\:{T}_{2}$ values for endplate under conventional $\:{T}_{2}$ mapping [19]. UTE-$\:{T}_{2}^{*}$ mapping values consistently correlated with $\:{T}_{2L}$ values. The difference between the moderate and mild damage groups was statistically significant (*p* < 0.005), demonstrating the bi-exponential $\:{T}_{2}^{*}$ model’s high sensitivity in CEP damage assessment.

Conversely, the $\:{T}_{2S}$ faction exhibited no statistically significant disparities among the three groups (*p* = 0.58), signifying that the proportion of bound to free water remained largely unaltered during cartilage endplate degeneration. This suggests that the changes in the water molecule microenvironment necessary to cause significant alterations in tissue structure have not yet occurred in mild CEP damage, which limits the ability of machine learning models to make early identifications [20]. Although the short $\:{T}_{2}^{*}$ fraction, a more direct surrogate of bound water proportion, did not differ significantly between groups, changes in the local microenvironment can lead to an increase in $\:{T}_{2S}$ without altering its fractional contribution. This suggests that $\:{T}_{2S}$ primarily reflects microstructural degeneration of the collagen matrix rather than gross changes in bound water content, which may explain the limited ability of quantitative parameters to detect very early CEP damage [21]. Therefore, to improve sensitivity in identifying the mild CEP damage stage, a combination of metabolic imaging, biochemical markers, and other microscopic parameters may be important for optimizing early diagnostic strategies [22].

Previous monoexponential UTE-$\:{T}_{2}^{*}$ studies reported CEP $\:{T}_{2}^{*}$ values ranging from 9.9 to 21.8 ms [5], which are higher than the $\:{T}_{2L}$ values observed in this study. These values are not directly comparable to the $\:{T}_{2L}$ component derived from bi-exponential modeling, as monoexponential fitting reflects a composite decay of multiple water compartments. In contrast, the bi-exponential model separates short and long $\:{T}_{2}^{*}$ components, with $\:{T}_{2L}$ representing the free water pool after removal of the rapidly decaying bound water signal. Within this framework, The increase in $\:{T}_{2L}$ observed in the mildly impaired group may be associated with early matrix loosening and partial disruption of the collagen-rich endplate structure, which enhances free water mobility. In contrast, the subsequent decrease in $\:{T}_{2L}$ in the moderately impaired group may result from more advanced structural damage, including collagen degradation, reduced tissue permeability, and partial replacement by fibrotic or calcified components, which restrict free water motion and shorten $\:{T}_{2L}$ relaxation times.

This study has several limitations. First, the data came from a single hospital with a limited sample size. Second, the $\:{T}_{2}^{*}$ time of the cartilage endplate is extremely short, so differences in $\:{T}_{2}$ measurements may result from different scanning equipment or parameter settings. Third, CEP regions were manually segmented, which may introduce observer-related variability despite the use of a standardized protocol and secondary review. However, manual delineation remains a practical approach for accurately localizing the thin and irregular morphology of the CEP in UTE imaging. Future studies will focus on developing automated or semi-automated segmentation methods based on deep learning to reduce manual intervention and potential mislabeling. Additionally, a limited number of echo times were used for bi-exponential T2* fitting. Although four echoes were acquired to estimate four model parameters, the analysis was primarily intended to characterize group-level differences in CEP microstructure rather than absolute voxel-wise relaxation constants. Future studies with denser echo sampling, particularly additional echo times below 1 ms, are warranted to further improve parameter identifiability and fitting robustness.

In conclusion, we assessed the significance of the UTE bi-exponential $\:{T}_{2}^{*}$ model in the evaluation of CEP degeneration. The UTE bi-exponential model sensitively reveals changes in endplate microstructure and composition. Although, $\:{T}_{2L}$ values can serve as an imaging marker for CEP degeneration and provide a new quantitative index for the clinical diagnosis and monitoring of disease progression, our research suggests that both LR and SVM models on multiple parameters of both the free water and bound water components are more efficient than conventional $\:{T}_{2}^{*}$ mapping. We anticipate that this can guide clinical decision-making and efficacy assessment.

## Data Availability

The datasets generated and/or analyzed during the current study are available from the corresponding author on reasonable request.
